# The Effect of Absorption-Enhancement and the Mechanism of the PAMAM Dendrimer on Poorly Absorbable Drugs

**DOI:** 10.3390/molecules23082001

**Published:** 2018-08-10

**Authors:** Juan Lu, Nannan Li, Yaochun Gao, Nan Li, Yifei Guo, Haitao Liu, Xi Chen, Chunyan Zhu, Zhengqi Dong, Akira Yamamoto

**Affiliations:** 1 Institute of Medicinal Plant Development, Chinese Academy of Medical Sciences, Peking Union Medical College, Beijing 100094, China; jlu@implad.ac.cn (J.L.); linan837@163.com (N.L.); 17801085365@163.com (Y.G.); 17801080758@163.com (N.L.); yfguo@implad.ac.cn (Y.G.); htliu@implad.ac.cn (H.L.); chenx_implad@163.com (X.C.); cyzhu@implad.ac.cn (C.Z.); 2 Research Center on Life Sciences and Environmental Sciences, Harbin University of Commerce, Harbin 150076, China; 3 Key Laboratory of Bioactive Substances and Resources Utilization of Chinese Herbal Medicine, Ministry of Education, Chinese Academy of Medical Sciences, Peking Union Medical College, Beijing 100094, China; 4 Department of Biopharmaceutics, Kyoto Pharmaceutical University, Kyoto 607-8414, Japan

**Keywords:** PAMAM dendrimer, fluorescein isothiocyanate-dextrans, absorption-enhancement, mechanism

## Abstract

The polyamidoamine (PAMAM) dendrimer is a highly efficient absorption promoter. In the present study, we studied the absorption-enhancing effects and the mechanism of PAMAM dendrimers with generation 0 to generation 3 (G0–G3) and concentrations (0.1–1.0%) on the pulmonary absorption of macromolecules. The absorption-enhancing mechanisms were elucidated by microarray, western blotting analysis, and PCR. Fluorescein isothiocyanate-labeled dextrans (FDs) with various molecular weights were used as model drugs of poorly absorbable drugs. The absorption-enhancing effects of PAMAM dendrimers on the pulmonary absorption of FDs were in a generation- and concentration-dependent manner. The G3 PAMAM dendrimer with high effectiveness was considered to the best absorption enhancer for improving the pulmonary absorption of FDs. G3 PAMAM dendrimers at three different concentrations were non-toxic to Calu-3 cells. Based on the consideration between efficacy and cost, the 0.1% G3 PAMAM dendrimer was selected for subsequent studies. The results showed that treatment with a 0.1% G3 PAMAM dendrimer could increase the secretion of organic cation transporters (OCTs), OCT1, OCT2, and OCT3, which might be related to the absorption-enhancing mechanisms of the pulmonary absorption of FDs. These findings suggested that PAMAM dendrimers might be potentially safe absorption enhancers for improving absorption of FDs by increasing the secretion of OCT1, OCT2, and OCT3.

## 1. Introduction

As a novel class of artificial macromolecules, polyamidoamine (PAMAM) dendrimers have shown excellent performance in biomedical applications due to their unique physical and chemical properties [1,2,3]. PAMAM dendrimers possess high water solubility, and they, as highly efficient absorption promoters, can easily penetrate through membranes. In addition, they can be used as carriers for different routes of drug administration systems [4,5,6,7].

Up to now, dendrimers have already attracted increasing attention for their applications in many fields, including supramolecular chemistry [8,9], electrochemistry [10], photochemistry [11], nanoparticle synthesis template [12,13], monomolecular membranes [14], curing agents in epoxy resin systems [15], drug delivery systems [16,17,18,19,20], and gene transfection [21,22] in biomedical fields. Among the abovementioned applications, the use of dendrimers as drug carriers in delivery systems has been of great interest [23].

As absorption enhancers, the safety of PAMAM dendrimers was an important factor to determine when deciding whether these enhancers were to be applied in clinical practice. Significantly, undesired or uncontrolled dendrimer interactions with blood components and host tissues can readily produce serious toxicity [24]. Cytotoxicity induced by surface positive charges of dendrimers, which were increased with higher generations and concentrations, has been previously reported [1,5,25]. Hemotoxicity is an undesired type of cytotoxicity of PAMAM dendrimers. It has been found that the cationic PAMAM dendrimers result in blood platelet activation, leading to prothrombotic effects [26,27]. PAMAM dendrimers also have a negative effect in the human neural pathways [28,29]. Furthermore, dendrimers are highly charged particles, and therefore, they agglomerate greatly, particularly on the cell membrane [30]. PAMAM dendrimers (G0–G3) were chosen as the absorption enhancers, because they have been proven in our previous study not to cause any mucosal damage to the lung tissues [5].

In order to improve the bioavailability of oral drugs, the research of absorption promoters is a hotspot in pharmaceutical preparations. Experimental results have shown that the mechanisms of absorption enhancers are non-specific, which can strengthen the fluidity of the cell membrane, promote the formation of membrane pores, reduce the viscosity of the mucous layer, and increase the permeability of the membrane. An absorption promoter can enhance pharmacodynamic ability, leading to a temporary change of the tight junctions between the epithelial cells.

The DNA microarray is now recognized as a useful device in molecular biology and is widely used to identify molecular mechanisms. Genome-wide analysis of gene expression by oligonucleotide microarray is a powerful molecular tool for the discovery of new genes involved in molecular processes. The DNA microarray is mainly used for the study of gene function, and thousands of large-scale and high-flux genes can be studied at the same time by gene chip, which may solve the problems of the traditional complex operation of nucleic acid blot hybridization and low detection efficiency. Through the design of different arrays, a specific analysis method can make the application of this technology have different values. Besides passive diffusion and bypass diffusion about the mechanisms of drug absorption, many of which are active vector-mediated transport, when the carrier protein is involved in drug transit, the expression levels of these proteins will be correspondingly altered. Therefore, we can understand the types and mechanisms of carrier proteins based on their change tendency.

In the present study, we mainly focused on two drug transporter families: solute carrier (SLC) and ATP-binding cassette (ABC). These two families have important functions in the processes of drug absorption and transport. Drug membrane transporters from SLC and ABC families play a fundamental role in such processes. We investigated the effect of G3 PAMAM dendrimer on organic cation transporters (OCTs: OCT1, OCT2, and OCT3) of the SLC family and P-glycoprotein (P-gp), which is the most important ABC protein encoded by ABCB1 (a multidrug resistance protein1 (MDR1)) gene [31].

Moreover, we comprehensively assessed the mechanism underlying the absorption-enhancing effects of PAMAM dendrimers, as a type of absorption promoter, at the animal, cellular, and molecular levels. Meanwhile, our data showed that PAMAM dendrimers were characterized by high efficiency, low toxicity, and clear mechanism of action. Taken together, our findings suggested that PAMAM dendrimers could be used as a novel class of absorption promoter.

## 2. Results

### 2.1. Absorption-Enhancing Effects of PAMAM Dendrimers on the Pulmonary Absorption of Fluorescein Isothiocyanate-Labeled Dextrans (FDs)

**Figure 1:**
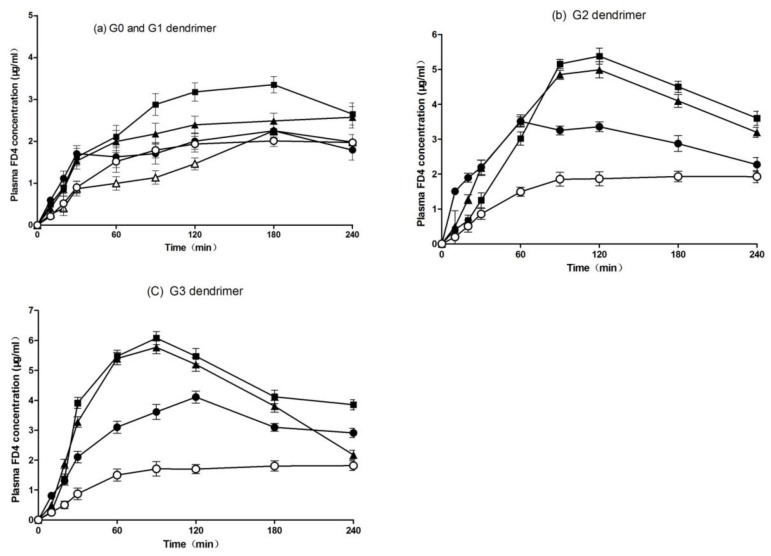
Plasma concentration-time profiles of fluorescein isothiocyanate-labeled dextrans 4 (FD4) after pulmonary administration with various concentrations of G0 and G1 polyamidoamine (PAMAM) dendrimers (**a**), the G2 dendrimer (**b**), or the G3 dendrimer (**c**) to rats. Each point represents the mean ± S.E. of 3–5 experiments. Keys: (**a**) Control (○), 0.5% (*w*/*v*) G0 (△), 0.1% (*w*/*v*) G1 (●), 0.5% (*w*/*v*) G1 (▲), 1% (*w*/*v*) G1 (■); (**b**) Control (○), 0.1% (*w*/*v*) G2 (●), 0.5% (*w*/*v*) G2 (▲), 1% G2 (*w*/*v*) (■); (**c**) Control (○), 0.1% (*w*/*v*) G3 (●), 0.5% (*w*/*v*) G3 (▲), 1% G3 (*w*/*v*) (■).

Figure 1 shows the plasma concentration–time profiles of FD4 after pulmonary administration of PAMAM dendrimers with different concentrations and generations. As shown in Figure 1a, the 0.1% (*w*/*v*) G1 and 0.5% (*w*/*v*) G0 PAMAM dendrimers did not enhance the absorption of FD4, while we observed a slight increase in the plasma concentration of FD4 when the 0.5% (*w*/*v*) and 1% (*w*/*v*) G1 PAMAM dendrimer were used as absorption enhancers.

Figure 1b shows the effect of the G2 PAMAM dendrimer on the pulmonary absorption of FD4. The plasma concentration of FD4 was significantly increased in the presence of the G2 dendrimer at all concentrations [0.1% (*w*/*v*), 0.5% (*w*/*v*), and 1% (*w*/*v*)]. The G2 dendrimer exhibited an absorption-enhancing effect in a concentration-dependent manner. The effect of the G3 PAMAM dendrimer on the pulmonary absorption of FD4 was also examined (Figure 1c). The G3 dendrimer at all concentrations [0.1% (*w*/*v*), 0.5% (*w*/*v*), and 1% (*w*/*v*)] significantly increased the plasma concentration of FD4 compared with the control group, while 1% (*w*/*v*) of the G3 PAMAM dendrimer displayed the greatest absorption-enhancing effect. Overall, the absorption-enhancing effect of the G3 PAMAM dendrimer was positively correlated with its concentration.

Table 1 summarizes the pharmacokinetic parameters (Cmax (peak concentration), Tmax (peak time), AUC (area under the concentration-time curve), and absorption enhancement ratio) after pulmonary administration of FD4 with various generations and concentrations of the PAMAM dendrimer. Moreover, the absorption enhancement ratios, which were calculated from the AUC values in the presence or absence of the PAMAM dendrimer, were 0.9, 1.3, 2.4, and 2.7 for the 0.5% (*w*/*v*) G0, 0.5% (*w*/*v*) G1, 0.5% (*w*/*v*) G2, and 0.5% (*w*/*v*) G3 PAMAM dendrimer, respectively. These findings suggested that the absorption-enhancing effects of the PAMAM dendrimer on the pulmonary absorption of FD4 were generation-dependent. Therefore, the absorption-enhancing effect of the PAMAM dendrimer could be ranked in an order as follows: G3 > G2 > G1 > G0. The G0 dendrimer had no absorption-enhancing effect on the pulmonary absorption of FD4 in this study. On the other hand, Table 1 also demonstrates the effects of the PAMAM dendrimer at different concentrations on the pulmonary absorption of FD4. We found that the absorption-enhancing effect of the PAMAM dendrimer on the pulmonary absorption of FD4 was increased as the concentration of the same generation of the PAMAM dendrimer was increased. The absorption enhancement ratios of the G3 dendrimer at concentrations of 0.1% (*w*/*v*), 0.5% (*w*/*v*), and 1% (*w*/*v*) were 2.0, 2.7, and 2.9, respectively. In addition, the PAMAM dendrimer with all generations and concentrations could decrease the Tmax of FD4, although some dendrimers did not increase the Cmax and AUC values of FD4 after their pulmonary absorption. These findings suggested that PAMAM dendrimers could increase the absorption rate of FD4 after pulmonary administration in rats.

**Figure 2:**
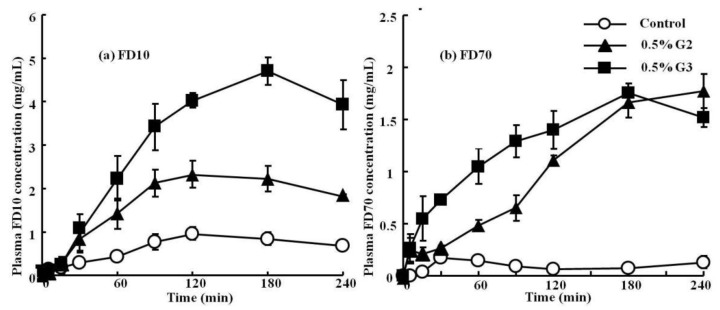
Plasma concentration–time profiles of FD10 (**a**) and FD70 (**b**) after pulmonary administration with the 0.5% G2 and G3 dendrimer to rats. Each point represents the mean ± S.E. of 3–5 experiments. Keys: Control (○); 0.5% G2 dendrimer (▲); 0.5% G3 dendrimer (■).

Figure 2 shows the plasma concentration profiles of FD10 and FD70 after pulmonary administration with the 0.5% (*w*/*v*) G2 and G3 PAMAM dendrimers. Table 2 summarizes the pharmacokinetic parameters (Cmax, Tmax, AUC, and absorption enhancement ratio) of these drugs after pulmonary administration in the presence or absence of the 0.5% (*w*/*v*) G2 and G3 PAMAM dendrimers. We found that the plasma concentration of FD10 was increased by the co-administration of the 0.5% (*w*/*v*) G2 and G3 dendrimers. Similarly, a significantly increased plasma concentration of FD70 was also observed by the co-administration of the 0.5% (*w*/*v*) G2 and G3 PAMAM dendrimers (Figure 2). Moreover, the Cmax and AUC of FD10 and FD70 were increased in the presence of the 0.5% (*w*/*v*) G2 and G3 dendrimers, suggesting that PAMAM dendrimers could increase the absorption of these drugs after their pulmonary administration. Collectively, the 0.5% (*w*/*v*) G2 and G3 PAMAM dendrimers could enhance the pulmonary absorption of macromolecular compounds, including FD10 and FD70.

### 2.2. In Vitro Toxicity

**Figure 3:**
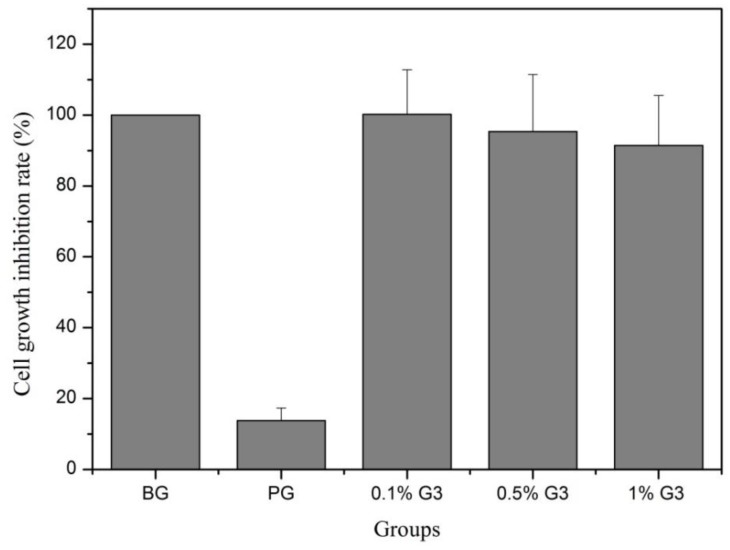
The cytotoxicity in vitro of different concentrations of G3 PAMAM.

To investigate the effect of the G3 PAMAM dendrimer on Calu-3 cells, the cell proliferation was assessed by 3-(4,5-dimethylthiazol-2)2,5-difeniltetrazolium bromide (MTT) assay. Figure 3 shows that the cell growth inhibition rate of the 0.1% G3 PAMAM dendrimer was 100.2 ± 12.6%. The toxic reaction was classified as grade 0. The cell growth inhibition rate of the G3 dendrimer at concentrations of 0.5% and 1% was 95.3 ± 16.1% and 91.4 ± 14.1%, respectively. The in vitro cytotoxicity was classified as grade 1. These results suggested that the G3 PAMAM dendrimer at three different concentrations was non-toxic to Calu-3 cells.

### 2.3. Absorption-Enhancing Effects of PAMAM Dendrimer on Cell Experiments of FD4

**Figure 4:**
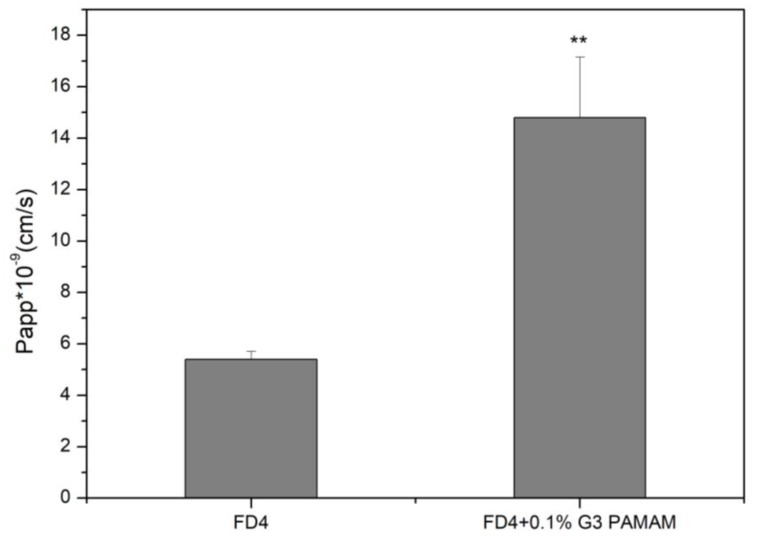
Transport of 5 mg/mL FD4 sulfate across Calu-3 cell layers, in the absence (control) and in the presence of the 0.1% G3 PAMAM dendrimer (Average ± SD, *n* = 6), (**) *p* < 0.01, compared with the control group.

FD4 transport was studied at a concentration of 5 mg/mL, and the flux for FD4 was shown in Figure 4. Data analysis suggested that FD4 containing the 0.1% G3 PAMAM dendrimer had significantly higher transport compared with the FD4 alone, suggesting an active uptake mechanism. The apparent permeability coefficient (Papp) was determined to further study the potential of active transport. Across Calu-3 cell monolayers, the Papp in the FD4 was (5.40 ± 0.30) × 10^−9^ cm/s, which was increased by 2.74-fold compared with (14.8 ± 2.35) × 10^−9^ cm/s in the FD4 containing the 0.1% G3 PAMAM dendrimer. This finding suggested the absorption-enhancing effects of PAMAM dendrimers on cell experiments of FD4.

### 2.4. Microarray Mechanical Testing

**Figure 5:**
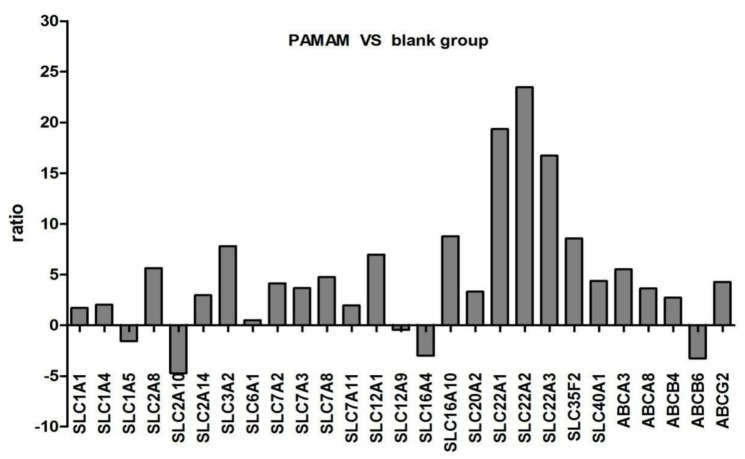
The gene list that represents the fold change on genes encoding SLC and ABC proteins in response to the 0.1% G3 PAMAM dendrimer.

GeneChip analysis revealed that a large number of genes were differentially regulated in response to sub-inhibitory concentrations of the 0.1% G3 PAMAM dendrimer. We analyzed the expressions of genes from the ABC and SLC superfamilies in the 0.1% G3 PAMAM dendrimer cell lines and control cell lines. Table 3 and Figure 5 summarize the alteration in the gene expression of the ABC and SLC superfamilies, respectively. Significant changes greater than twofold and less than 0.5-fold were considered for the evaluation of their contributions. The genes with expression alteration between twofold and 0.5-fold were considered as ‘not significant (NS)’ in constructing gene lists. From 350 SLC genes, we found that the expressions of 20 genes were significantly changed. In the expression analysis of 20 genes, three genes were significantly increased by over 10-fold, including SLC22A2, SLC22A1, and SLC22A3. The expressions of five ABC genes were also changed, including four upregulated ones.

### 2.5. Mechanism Analysis of Absorption-Enhancing Effects

**Figure 6:**
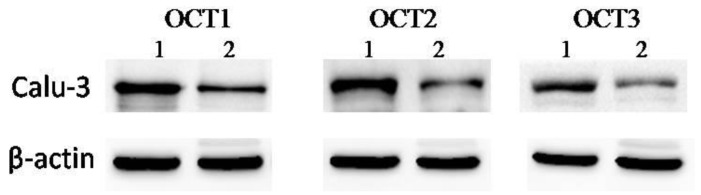
Western blot analysis of organic cation transporters (OCTs) production. Expression of OCTs in Calu-3 cell layers, cultured for 14 days by western blot analysis (Line 1, 0.1% G3 PAMAM dendrimer treatment; line 2, positive control). The data shown are from a single representative experiment and were reproduced three times.

Western blotting analysis was performed to detect the expressions of OCT1, OCT2, and OCT3 at the protein level. As exoproteins are principally secreted during post-exponential growth, sample cell lines were cultured in the presence of the 0.1% G3 PAMAM dendrimer. Figure 6 shows that treatment of the 0.1% G3 PAMAM dendrimer increased the secretion of OCT1, OCT2, and OCT3.

**Figure 7:**
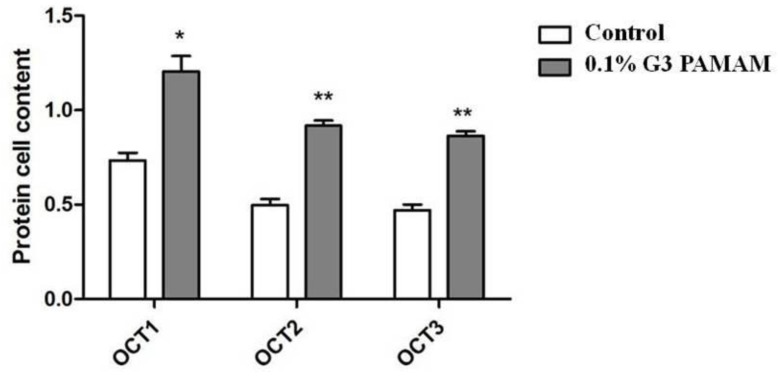
PAMAM dendrimer induces the enhancement of OCT proteins. (**) *p* < 0.01, (*) *p* < 0.05, compared with the control group.

Calu-3 cells were incubated in the presence of the absence of the 0.1% PAMAM dendrimer for 2 h. Total cell extracts were analyzed by sodium dodecyl sulfate-polyacrylamide gel electrophoresis (SDS-PAGE) and immunoblotting. Figure 7 summarizes the statistical analysis.

**Figure 8:**
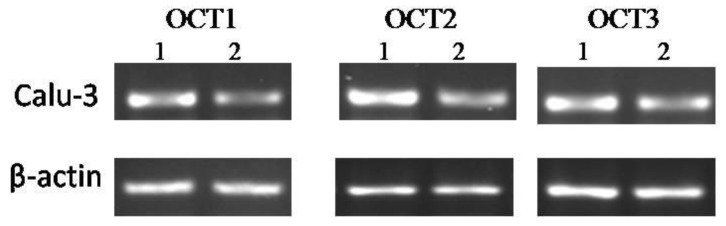
PCR analysis of OCTs production. Expression levels of mRNA encoding for human organic cation transporters (hOCTs) in Calu-3 cells (Line 1, 0.1%G3 PAMAM dendrimer treatment; line 2, positive control). The data shown are from a single representative experiment and were reproduced three times.

**Figure 9:**
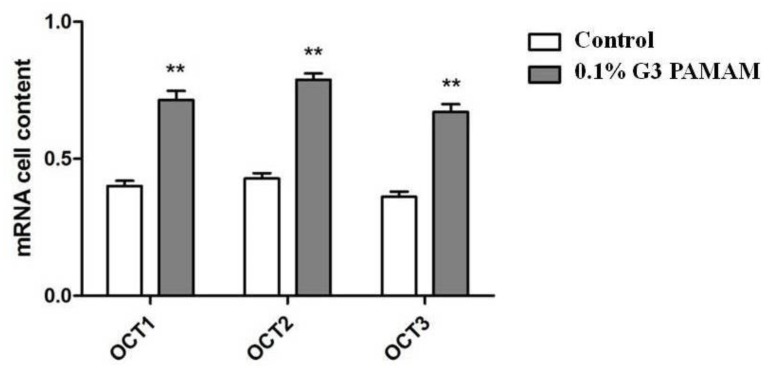
PAMAM dendrimers enhance the level of mRNA in OCTs. (**) *p* < 0.01, compared with the control group.

The expressions of human organic cation transporters (hOCTs), hOCT1, hOCT2, and hOCT3, at the mRNA level in Calu-3 cell lines were detected by RT-PCR. Figure 8 shows that the expressions of the abovementioned genes were increased in Calu-3 cell lines treated with the 0.1% G3 PAMAM dendrimer compared with the control group. Figure 9 reveals that the 0.1% PAMAM dendrimer enhanced the expressions of OCTs at the mRNA level, and such increase was sensitive.

## 3. Discussion

Although there are some reports regarding the absorption-enhancing effect of polyamine alone or the synergistic absorption-enhancing effect together with bile salts for improving the intestinal absorption of drugs [32,33], few studies have been carried out to examine the effects of PAMAM dendrimers on the pulmonary absorption of poorly absorbable drugs [34]. In this study, we examined the absorption-enhancing effects of PAMAM dendrimers on the pulmonary absorption of FDs.

Our findings demonstrated that PAMAM dendrimers had great absorption-enhancing effects on the pulmonary absorption of FDs. We found that the pulmonary absorption of FDs was significantly enhanced by the PAMAM dendrimers with various generations and concentrations, and the absorption-enhancing effects were in a generation- and concentration-dependent manner. These findings were in agreement with our previous data [5], showing that PAMAM dendrimers can improve the pulmonary absorption of insulin and calcitonin and enhance the pulmonary absorption both in a generation- and concentration-dependent manner. The absorption enhancement ratios of the G3 dendrimer at concentrations of 0.1% (*w*/*v*), 0.5% (*w*/*v*), and 1% (*w*/*v*) for the pulmonary absorption of FD4 were 2.0, 2.7, and 2.9, respectively. In addition, the 0.5% (*w*/*v*) G2 and G3 dendrimers could also enhance the pulmonary absorption of macromolecular compounds, including FD10 and FD70. The enhancement ratio of FD70 in the presence of the 0.5% G3 PAMAM dendrimer was more than 13.9 times higher compared with the control group. Therefore, the results indicated that PAMAM dendrimers were much more effective for improving the pulmonary absorption of FDs compared with conventional absorption enhancers [35].

To investigate the absorption-enhancing effects of PAMAM dendrimers in vitro, the Calu-3 cell monolayers were exposed to FD4 solution in the presence or absence of the 0.1% G3 PAMAM dendrimer (*w*/*v*). Compared with FD4, the Papp in the FD4 containing the 0.1% G3 PAMAM dendrimer was increased by 2.74-fold. The results showed that the PAMAM dendrimers had absorption-enhancing effects on cell experiments of FD4, which was consistent with the in vivo findings.

The mechanism by which PAMAM dendrimers enhance the pulmonary absorption of FDs remains largely unexplored. Two meaningful aspects of the proposed mechanism need to be discussed in more detail.

In the present work, we examined the expressions of ABC and SLC genes. Among SLC genes, 16 genes were upregulated and three were downregulated. Moreover, three genes, including SLC22A1, SLC22A2, and SLC22A3, were significantly upregulated (over 10-fold). Based on the expression profiles, our results provided a preliminary insight into the relationship between the absorption-enhancing effect and expressions of membrane transporters. OCTs exist in the SLC22 family, which belongs to the major facilitator superfamily. The SLC22 family plays a pivotal role in drug absorption and excretion. This family can be divided into various subgroups according to substrates and transport mechanisms. OCT1, OCT2, and OCT3 mediate hepatic, renal, and biliary excretion of many cationic drugs, respectively [36]. ABC is associated with gut mucosa among the important efflux transporters. They represent the largest family of transmembrane proteins. These proteins bind to and use the energy of ATP to drive the transport of various molecules across all cell membranes. P-gp is the most important protein in the ABC family, which can transfer cationic or neutral substances, and it is an important contributor to the body’s resistance [37]. The expressions of these transporters may be related to the enhancement of drug oral bioavailability. Western blotting analysis and RT-PCR were performed to detect the expressions of OCT1, OCT2, and OCT3. The results showed that treatment with the 0.1% G3 PAMAM dendrimer increased the secretion of OCT1, OCT2, and OCT3. Therefore, the absorption-enhancing effect of the PAMAM dendrimers on FDs was achieved by modulating the expressions of OCT1, OCT2, and OCT3.

Another possible mechanism is that PAMAM dendrimers enhanced the pulmonary absorption of FDs by changing the molecules’ negative surface charges to positive and increasing their permeability across the cell membranes. Because the surface of the mucus membrane contained negatively charged sialic acids, the coulombic repulsion effect generated between the negatively charged cell membrane and the negatively charged drug perhaps contributed in part to the poor absorbability of FDs (in the absence of PAMAM dendrimers) from the alveolar epithelium. The change of charge to positive by the addition of PAMAM dendrimers may diminish the coulombic repulsion effect and consequently increase drug transport across the alveolar epithelium [5,38,39].

## 4. Materials and Methods

### 4.1. Materials

Fluorescein isothiocyanate-labeled dextran (FDs) with an average molecular weight of 4400 (FD4), 9100 (FD10), and 71,600 (FD70), as well as PAMAM G0, G1, G2, and G3 dendrimers (20 wt. % in methanol 1 g) were purchased from Sigma-Aldrich Chemical Co. (St. Louis, MO, USA). Dulbecco’s Modified Eagle′s medium (DMEM) and dimethyl sulfoxide (DMSO) were obtained from Sigma Aldrich (Sydney, Australia).

A bicinchoninic acid (BCA) protein assay kit was provided by Pierce Biotechnology Inc. (Rockford, IL, USA). Phosphate-buffered saline (PBS), Hank′s Balanced Salt Solution (HBSS), BenchMarkTM Pre-Stained Protein Ladder, and all Western blotting regents were supplied by Gibco, Invitrogen (Sydney, Australia). The chemiluminescence kit was purchased from Roche Diagnostics Australia Pty. Ltd. (Castle Hill, Australia). Sodium dodecyl sulfate (SDS) was obtained from J.T. Baker (Phillipsburg, NJ, USA).

Organic cation antibodies (OCT1, OCT2 and OCT3) (rabbit polyclonal IgG) were purchased from Abcham PLC (Cambridge, UK). Horse radish peroxidase (HRP)-conjugated secondary antibodies (goat anti-rabbit IgG or goat anti-rat IgG) were obtained from Cell Signaling Technology, Inc. (Danvers, MA, USA). Antibodies against β-actin (rat polyclonal IgG) were purchased from Abcham PLC (Cambridge, UK). Transwell cell culture inserts (1.12 cm^2^ polyester, 0.4 μm pore size) and black 96-well plates were supplied by Corning Costar (Cambridge, MA, USA). All cell culture consumables were provided by Sarstedt (Adelaide, Australia). All other chemicals and solvents were of analytical grade unless otherwise indicated.

### 4.2. Preparation of Drug Solution

Dosing solutions containing model drugs were prepared in isotonic phosphate buffer (PBS) at pH 7.4 to yield a final concentration of 100 mg/mL (FD4, FD10) and 200 mg/mL (FD70), respectively. In certain experiments, various concentrations (0.1%, 0.5%, 1% (*w*/*v*)) of different generations (G0, G1, G2, and G3) of PAMAM dendrimers as absorption enhancers were added to different dosing solutions, and pH in these solutions was adjusted to 7.0–8.0, respectively.

### 4.3. Analytical Methods

The pulmonary absorption of FDs was estimated by measuring the concentrations of FDs in plasma. For the determination of the FD concentrations, 100 μL plasma was collected and added to an equal volume of 10% (*w*/*v*) Triton X-100. The fluorescence intensity of these samples was measured by a spectrofluorometer (Spectrafluor Plus, TECAN, Zürich, Switzerland) at an excitation wavelength of 485 nm and an emission wavelength of 535 nm.

### 4.4. Absorption-Enhancing Effects of PAMAM Dendrimers on the Pulmonary Absorption of FDs

Animal experiments were carried out in accordance with the guidelines of the Animal Ethic Committee at Kyoto Pharmaceutical University. Male Wister rats, weighing 200–250 g, were fasted for 12 h before the experiments, while free access to water was given to the animals. The rats were anesthetized by intraperitoneal injection of sodium pentobarbital solution at a dose of 50 mg/kg. A surgical operation for the in vivo pulmonary absorption study was performed according to a previously described method [40,41]. Briefly, the rat was secured on an animal board, and then, the trachea was exposed through a longitudinal incision along the ventral aspect of the neck. The trachea was then cut transversely, halfway through, between the fourth and fifth tracheal rings caudal to the thyroid cartilage. A section of polyethylene tubing (inside diameter, 1.5 mm, outside diameters, 2.5 mm) of 2.5 cm in length, which served as a tracheal cannula, was caudally inserted through the tracheal incision for a distance of 0.6 cm, by which 1.9 cm of the cannula protruded from the trachea. The body temperature of the animals was maintained at 37 ± 1 °C with a 40 W incandescent heat lamp and a reflector suspended over the animal at a distance of about 25 cm during the experiment. Subsequently, 100 μL of drug solution at a temperature of 37 °C was injected into the lungs through the obtuse needle of a calibrated 250 μL syringe. For the injection, the needle was inserted through the tracheal cannula to a depth of 2.5 cm below the tracheal incision. At this distance of injection, the tip of the syringe needle was located 1–2 mm above the tracheal bifurcation. Then, the solution was injected over a period of 1–2 s, with the rat being maintained at an angle of 80°. Immediately thereafter, the tubing was completely withdrawn, and the animal was returned to an angle of 10° at 45 s after administration. The jugular vein of the anesthetized rats was exposed using surgical forceps. Heparinized syringes were inserted into the jugular vein, and blood samples (0.2 mL) were collected at predetermined time intervals until up to 240 min. Samples were immediately centrifuged at 12,000 rpm for 5 min to obtain the plasma fraction (100 μL), which was then kept on ice prior to further analysis.

### 4.5. In Vitro Toxicity

#### 4.5.1. Cell Culture and Cell Lines

Calu-3 cells were maintained in 25 cm^2^ flasks in complete DMEM supplemented with 10% (*v*/*v*) FBS, 1% (*v*/*v*) non-essential amino acid solution, 1% (*v*/*v*) L-glutamine solution, penicillin (100 U/mL), and streptomycin (100 U/mL) at 37 °C in a humidified atmosphere of 95% air and 5% CO_2_. The culture medium was refreshed every 2 days.

When the cells reached 80% confluence, the cells were seeded onto a 12 TranswellTM board, and the culture medium was refreshed every day. In general, the cultured cell monolayers had a transepithelial electrical resistance (TEER) value. When such value was greater than 500 Ω, the cells were used in following studies. Cells cultured in the absence of drugs were used as controls, while the 0.1% G3 PAMAM dendrimer cell lines were established by maintaining the cells in the presence of drugs in a humidified atmosphere of 95% air and 5% CO_2_ at 37 °C for 2 h.

#### 4.5.2. MTT Assay

In vitro toxicity was determined by an MTT assay. Briefly, Calu-3 cells (5 × 10^4^/mL) were seeded into a 96 well tissue culture plate with 100 μL per well, and edge plates were filled with sterile PBS as controls. The cells were maintained in a humidified atmosphere of 95% air and 5% CO_2_ at 37 °C for 24 h. The medium was aspirated. Then, 100 μL of the PAMAM solution (0.1%, 0.5%, and 1% G3 PAMAM dendrimers) was added to each well; the blank control group (BG) and positive control group (3% Triton X-100, PG) were set, which were maintained for 2 h. Then, 10 μL of 5 mg/mL MTT reagent was added to each well, and the plate was incubated at 37 °C for 4 h. The cells were collected by centrifugation at 500 g for 10 min. The cell pellet was resuspended in 150 μL DMSO and incubated at room temperature for 10 min. The OD570 nm (A570) was determined using a microplate reader (Tecan, Austria). The viability and number of cells were represented by the OD570 nm value. The cell growth inhibition rate was calculated according to the formula as follows:

(A570 of control cells − A570 of treated cells) × 100%/A570 of control cells.(1)

### 4.6. Effects of 0.1% G3 PAMAM Dendrimers on Cell Experiments of FD4

#### 4.6.1. FD4 Transport Study

All experiments were conducted using Calu-3 cell monolayers between days 18–21 of the cell culture. FD4 transport was assessed at a concentration of 5 mg/mL to establish the directional flux.

Before each study, the culture medium was aspirated from both compartments, and the monolayer was left and equilibrated with warm HBSS for 30 min (in an incubator at 37 °C). Then, the TEER value was measured and recorded. For the study, both sides of the filter inserts were washed twice with HBSS, and then, the HBSS was removed from the apical compartment and replaced with 0.5 mL FD4 solution in the presence or absence of the 0.1% G3 PAMAM dendrimer (*w*/*v*). Subsequently, 200 μL of basal sample was collected at predetermined time points (10, 20, 30, 60, 90, and 120 min) and replaced with 200 μL of fresh pre-warmed HBSS. After 2 h, the TEER value was measured and recorded again. All experiments were conducted in triplicate, and all the samples collected were analyzed using a spectrofluorometer (Spectrafluor Plus, TECAN, Switzerland). Excitation and emission wavelengths were set at 485 nm and 535 nm, respectively.

#### 4.6.2. Calculation of Apparent Permeability

The apparent permeability coefficient Papp (cm·s^−1^) of FD4 across the Calu-3 monolayer was calculated according to the following equation:

(2) $$P_{\mathrm{app}}=\frac{dC}{dt}÷(A\times C_{0})$$

where (*dC*/*dt*) is the flux of drug in mol sec^−1^, *A* is the surface area of the Transwell in cm^2^, and *C*_0_ the initial concentration in donor compartment in mol·cm^−3^.

### 4.7. Microarray

#### 4.7.1. RNA Isolation and cDNA Labeling

Immediately before harvesting the Calu-3 cell for RNA isolation, the samples were treated with the RNA protect reagent to minimize RNA degradation. The cells were collected by centrifugation and stored at −80 °C. RNA was isolated from the 0.1% G3 PAMAM dendrimer cell lines and the control cell lines using a RNeasy Mini kit (QLAGEN) according to the manufacturer′s instructions. RNA was then re-purified with NucleoSpinORRNA clean-up (MACHEREY-NAGEL, Düren, Germany). The absorbance values (260, 280 nm) were measured for RNA quantification by spectrophotometry. RNA intactness was checked by 1% *w*/*v* denaturing agarose gel electrophoresis. cDNA was synthesized in two steps named first strand synthesis and second strand synthesis. RNA was reverse-transcribed into cDNA, which was subsequently labeled.

#### 4.7.2. GeneChip Hybridization and Scanning

Labeled cDNA samples from independent RNA preparations were hybridized into genome arrays according to the manufacturer′s instructions for antisense prokaryotic arrays. The hybridization procedure was conducted at 42 °C for 14 h. Capital Bio Lux Scan 10KA Imaging Station was used for scanning the arrays.

Preliminary analysis of the scanned chips was performed using Microarray Analysis Lux Scan 3.0 (Capital-Bio) software. The quality of gene expression data was assessed according to quality control criteria provided by the software. Gene expression was examined using GeneChip Operating Software, and a table of genes with significant changes was prepared. Significance Analysis of Microarrays (SAM) software was used to identify the genes differentially expressed in the 0.1% G3 PAMAM dendrimer cell lines compared with control cell lines. The false discovery rate (FDR) significance level was <5%.

### 4.8. Western Blotting Analysis

#### 4.8.1. Protein Extraction

The 0.1% G3 PAMAM dendrimer cell lines and control cell lines were lysed in an ice-cold radio-immunoprecipitation assay (RIPA) lysis buffer (pH 7.4) containing 50 mM Tris, 150 mM NaCl, 1% Triton X-100, 1% sodium deoxycholate, 0.1% SDS, sodium orthovanadate, sodium fluoride, and ethylenediaminetetraacetic acid (EDTA)for 30 min. Then, the cells were sonicated for 20 s and centrifuged at 12,000× *g* for 5 min at 4 °C.

#### 4.8.2. Protein Quantitation

The protein concentration of the cell lysate was determined using a Quant-iT Protein Assay BCA.

#### 4.8.3. Polyacrylamide Gel Electrophoresis

Equal amounts of proteins (30 μg) were subjected to sodium dodecyl sulfate-polyacrylamide gel electrophoresis (SDS-PAGE) on a 10% gel and then electrophoretically transferred onto nitrocellulose membranes. The membranes were blocked with 5% fat-free milk for 3 h at room temperature and then incubated with appropriate primary antibodies against human OCT1, OCT2, OCT3, and β-actin at 4 °C overnight. Following three washes with Tris-buffered saline (TBS), the blots were incubated with the HRP-conjugated secondary antibody (1:1000) at room temperature for 1 h. The blots were washed by TBS three times.

#### 4.8.4. Gel Imaging

The immunoreactive bands were detected using the enhanced chemiluminescence method.

### 4.9. Quantitative RT-PCR

Quantitative real-time reverse transcription (RT)-PCR was used to verify the microarray results. Aliquots of the RNA preparations from the 0.1% G3 PAMAM dendrimer-treated and control samples used in the microarray experiments were saved for quantitative real-time RT-PCR. Total RNA was isolated from cells using the Trizol method. To remove the genomic DNA, extracted RNA was treated with RNase-free DNase I (Ambion, Austin, TX, USA) at 37 °C for 20 min. The samples were either used immediately or stored at −80 °C. The concentration and purity of isolated RNA were determined by the NanoDrop method. RNA was reversely transcribed into cDNA using the Takara RNA PCR kit (AMV) Ver. 3.0 (Takara, Kyoto, Japan) according to the manufacturer′s instructions. The PCR reactions were performed in a 25 μL reaction system with specific primers and cycles (Table 4) on a 7000 real-time PCR system using a SYBR green universal PCR master mixture from Applied Biosystems. All the samples were analyzed in triplicate and normalized against 16S rRNA expression. The relative expression levels were determined by the ddCt method described in Applied Biosystems User Bulletin No. 2.

### 4.10. Statistical Analysis

The experimental data were analyzed using the SPSS 12.0 statistical software (Chicago, IL, USA). The results were expressed as mean ± S.E. An unpaired Student’s *t*-test was used to test the significance between drug and control groups. A *p* value of less than 0.05 was considered to be statistically significant. When an additional comparison was required, a one-way ANOVA (with Tukey′s post-hoc analysis) was utilized.

## 5. Conclusions

In conclusion, PAMAM dendrimers could improve the pulmonary absorption of poorly absorbable drugs, FDs. The absorption-enhancing effects were in a generation- and concentration-dependent manner. The mechanism was also investigated by analyzing the alteration in expression levels of ABC and SLC genes. The absorption-enhancing effect of the PAMAM dendrimer on FDs was achieved by regulating the expressions of OCT1, OCT2, and OCT3. These findings will provide useful information for the pulmonary absorption of poorly absorbable drugs, including peptide and protein drugs.

## Figures and Tables

**Table 1:** Pharmacokinetic parameters of FD4 after pulmonary administration with different concentrations and generations of the PAMAM dendrimer to rats.

	Cmax (μg/mL)	Tmax (min)	AUC_0__–__120_ (μg∙min/mL)	E. Ratio
Control	2.1 ± 0.2	170.0 ± 43.6	374.3 ± 36.3	1.0
0.5% G0	2.2 ± 0.2	170.0 ± 43.6	327.5 ± 23.2	0.9
0.1% G1	2.4 ± 0.3	120.0 ± 34.6	398.0 ± 62.3	1.1
0.5% G1	2.9 ± 0.4 *	127.5 ± 18.9	503.8 ± 51.8 *	1.3
1% G1	3.6 ± 0.4 *	135.0 ± 26.0	611.3 ± 68.3 **	1.6
0.1% G2	3.6 ± 0.3 *	80.0 ± 20.0	73.7 ± 30.1 **	1.8
0.5% G2	5.0 ± 0.4 **	90.0 ± 17.3	894.2 ± 55.7 **	2.4
1% G2	5.5 ± 0.5 **	120.0 ± 0.0	921.8 ± 106.6 **	2.5
0.1% G3	4.4 ± 0.2 **	100.0 ± 10.0	728.5 ± 43.6 **	2.0
0.5% G3	6.0 ± 0.4 **	90.0 ± 13.5 *	996.2 ± 74.4 **	2.7
1% G3	7.0 ± 0.5 **	102.0 ± 12.0	1103.0 ± 55.9 **	2.9

Note: The results are expressed as the mean ± S.E. of 3–5 experiments. (**) *p* < 0.01, (*) *p* < 0.05, compared with the control group. Cmax: peak concentration; Tmax: peak time; AUC_0__–__120_: area under the concentration-time curve from zero to 120 min; E. Ratio: absorption enhancement ratio.

**Table 2:** Pharmacokinetic parameters of FD10 and FD70 after pulmonary administration with the 0.5% (*w*/*v*) G2 and G3 dendrimer to rats.

	Cmax (μg/mL)	Tmax (min)	AUC0→12 (μg·min/mL)	E. Ratio
FD10				
Control	1.1 ± 0.1	150.0 ± 26.9	160.8 ± 16.9	1.0
0.5% G2	2.3 ± 0.3	130.0 ± 26.5	422.5 ± 46.4 **	2.6
0.5% G3	4.8 ± 0.2	130.0 ± 26.5	777.7 ± 11.0 **	4.8
FD70				
Control	0.2 ± 0.0		22.2 ± 2.4	1.0
0.5% G2	1.8 ± 0.2	200.0 ± 20.0	247.0 ± 13.7 **	11.1
0.5% G3	1.8 ± 0.1	180.0 ± 0.0	309.1 ± 22.8 **	13.9

The results are expressed as the mean ± S.E. of 3–5 experiments. (**) *p* < 0.01, compared with the control group.

**Table 3:** The gene list that represents the fold change on genes encoding solute carrier (SLC) and ATP-binding cassette (ABC) proteins in response to the 0.1% G3 PAMAM dendrimer. NS: not significant.

Gene Symbol	Up/Downregulation	Gene Symbol	Up/Downregulation
SLC1A1	NS	ABCA3	5.5290
SLC1A4	2.0397	ABCA8	3.6258
SLC1A5	−1.5712	ABCB4	2.7293
SLC2A8	5.6435	ABCB6	−3.2718
SLC2A10	−4.7236	ABCG2	4.2827
SLC2A14	2.9663		
SLC3A2	7.8088		
SLC6A1	0.5		
SLC7A2	4.1319		
SLC7A3	3.6651		
SLC7A8	4.7563		
SLC7A11	NS		
SLC12A1	6.9684		
SLC12A9	−0.4557		
SLC16A4	−3.0071		
SLC16A10	8.7864		
SLC20A2	3.3255		
SLC22A1	19.3628		
SLC22A2	23.4791		
SLC22A3	16.7368		
SLC35F2	8.5638		
SLC40A1	4.3789		

**Table 4:** Specific primer sequences and conditions for RT-PCR analysis.

Gene	Forward/Reverse Primer	Conditions	Product Size
LC22A1	F:CTGAGGGAGACATTGCACCT	95 °C 30 s	161 bp
	R:CGACAGCAGGCATAAGATGA	55 °C 30 s	
		72 °C 1 min	
LC22A2	F:CCGGATGTGGAACCTTATTGT	95 °C 30 s	179 bp
	R:CGGTGGTCTGTTGGATGGT	58 °C 30 s	
		72 °C 1 min	
SLC22A3	F:GTCACCTTCGCCTTCCTCTT	95 °C 30 s	242 bp
	R:CAGCTGAGAGCGCTAGTGG	60 °C 30 s	
		72 °C 1 min	
ACTB	F:AAACTGGAACGGTGAAGGTG	95 °C 30 s	171 bp
	R:AGAGAAGTGGGGTGGCTTTT	60 °C 30 s	
		72 °C 1 min	
